# A study of knowledge, attitudes, and practices of primary care physicians toward anticoagulant therapy in patients with non-valvular atrial fibrillation in Shanghai, China

**DOI:** 10.1186/s12875-020-01236-4

**Published:** 2020-08-15

**Authors:** Shasha Ye, Tianhao Wang, Arthur Liu, Ying Yu, Zhigang Pan, Jie Gu

**Affiliations:** 1 Department of Family Medicine, Jiahui International Hospital, No Guiping Rd, Shanghai, China; 2 Department of General Practice, Zhongshan Hospital, Fudan University, No 111 Yixueyuan Rd, Shanghai, China

**Keywords:** Non-valvular atrial fibrillation, Anticoagulant therapy, Primary care physician, Knowledge-attitudes-practices

## Abstract

**Background:**

As a large number of Community Health Service (CHS) centers in China face the majority of patients with non-valvular atrial fibrillation (NVAF), primary care physicians (PCPs) play a primary role in the prevention of embolization. Therefore, an awareness of anticoagulant management in patients with NVAF must be brought into focus among PCPs in China. This study investigated PCPs’ knowledge, attitudes, and practices toward anticoagulant therapy in patients with NVAF, to help them understand their shortcomings regarding oral anticoagulant (OAC) therapy in preventing embolization.

**Method:**

This was a cross-sectional observational study of 462 PCPs in CHS centers across Shanghai. We used a self-administered questionnaire to collect data from September to December 2017. A stratified random cluster sampling was adopted in the 90 CHS centers with the family medicine residency program.

**Result:**

Among 462 participants, 69.3% (320/462) of females received a medical bachelor’s degree and over 50% of participants had more than 10 years of work experience. Each section for knowledge, attitude, and practice were categorized as poor (≤39.0%), fair (40.0–69.0%), and good (≥70.0%). The level of knowledge of OAC therapy for patients with NVAF among PCPs was insufficient in over half (75.8%) of the participants. The majority (89.8%) of PCPs had a positive attitude and 68.0% had modest performance in the anticoagulant management of patients with NVAF.

**Conclusions:**

The knowledge and behaviors of PCPs were insufficient for OAC therapy to prevent embolization in patients with NVAF. The study also revealed that there is good potential for PCPs’ educational interventions to positively impact the care of patients with NVAF.

## Background

Atrial Fibrillation (AF) is the most common type of cardiac arrhythmia, especially in the elderly, and its prevalence increases with age [1]. According to the 2010 Global Burden of Disease Study, the number of patients with AF worldwide was 33 million, of which more than 13% were over 80 years of age [2]. The incidence of stroke in patients with AF is six times higher than those without AF, due to atrial contractile dysfunction and mural thrombosis formation [3, 4]. The burden of AF and associated strokes in China have increased significantly in recent years. The prevalence of AF has grown 20-fold, while the prevalence of AF-associated stroke has increased 13-fold in the past 11 years [1, 5]. Nearly 10 million patients with AF live in China, and NVAF is the most common type of AF (~ 62.5%) [1]. More than 24.8% of patients with AF in China have suffered ischemic strokes [3], and patients with AF with stroke are generally characterized by high morbidity, mortality, disability, and recurrence rates. These features make anticoagulant therapy a high priority for stroke-prevention strategies of AF [6, 7].

Substantial research shows that oral anticoagulant (OAC) therapy can greatly reduce the risk of stroke in patients with AF, and reduce the relative risk of ischemic stroke by about 62% [3, 8]. However, the current situation to OAC therapy in patients with NVAF in China is unsatisfactory, particularly in primary care settings [3]. In 2002, one Chinese study suggested that only 2% of patients with NVAF were on OAC therapy [3, 9], while another study found that less than 3% of 224 patients with AF were on such therapy [6]. Additionally, a mere 11.2% of patients with AF in China were on OAC therapy from 2008 to 2011, according to the RE-LY study [10, 11].

The standardization of OAC therapy in NVAF management is still in the initial stages in China [6]. China has not established OAC therapy guidelines to instruct physicians how to manage patients with NVAF. Moreover, doctors from different regions and hospitals lack consistency when providing advice to NVAF patients regarding OAC use [6].

To improve the prognoses of patients with NVAF, it is important to strengthen the use of OAC therapy in the long-term management of NVAF, and actively follow-up with continuous health education programs [6, 12]. CHS centers are indispensable due to their vast coverage, accessibility and convenience for disease prevention [13]. PCPs in CHS centers play vital roles in the prevention of stroke related to AF. The diagnosis and treatment of NVAFs are not standardized in most CHS centers [13, 14], and an awareness of anticoagulant management in patients with NVAF must be brought into focus among PCPs in China.

To date, inadequate studies and analyses of the factors influencing PCPs’ knowledge, attitude and practice (KAP) of anticoagulant management in patients with NVAF have been performed. Thus, we aimed to investigate the KAPs of PCPs in CHS centers in Shanghai and to identify the factors influencing such KAPs.

## Methods

### Sample size

A total of 460 participants were enrolled in the study, allowing for a 10% non-response rate, a 10% invalid return rate, and limited research fees.

### Study setting and population

Our cross-sectional survey adopted stratified random cluster sampling among PCPs working in CHS centers in Shanghai from September to December 2017. The ratio of urban, urban-rural, and rural CHS centers in 245 centers, including 90 CHS centers with family medicine residency programs was 80:84:81, which is equal to 1:1:1, respectively. We used the random number table to choose CHS centers in a 1:1:1 ratio from the 90 CHS centers.

The inclusion criteria for participants were: 1) worked in one of 90 CHS centers from September to December 2017; 2) registered as a General Practitioner; 3) agreed to participate in the study, and was willing to provide written informed consent.

### Questionnaires

We used a self-administered questionnaire that was composed of three parts (seen in supplementary 1) to collect data from PCPs in the Shanghai community. The 52-item KAP questionnaire was developed and validated by the Delphi technique described by Shasha Ye et al. [15] The clarity and intelligibility of each item of the KAP questionnaire were pretested.

Each score within the Knowledge section of the KAP questionnaire was based on the following: a score of one was given for one correct answer; a score of zero was given to incorrect or uncertain answers. A five-point Likert scale from 1 (completely disagree) to 5 (completely agree) was used with a neutral midpoint for each question of the A section, except item D4 whose answer was opposite to the others. A four-point Likert scale from 1 (never) to 4 (always) was used for each question in section P. According to a similar study, each section for knowledge, attitude, and practice were categorized as poor (≤39.0%), fair (40.0–69.0%), and good (≥70.0%) [16].

### Data collection

Questionnaires required approximately 10–20 min for PCPs to complete. All data collection teams participated in a training workshop. The investigators who collected the data did not participate directly in the management of the PCPs. The questionnaires were requested to be completed independently on site and collected on time. Questionnaires with repetitive patterns of answers or those in which all responses were the same were excluded following manual assessments. Data collection was conducted from September to December 2017. Following the collection of questionnaires, all data were entered into an EpiDate3.1 Database.

### Data analysis

Descriptive analyses were conducted using SPSS statistical package, version 22.

Frequency distributions, percentages, means, and standard deviations were used to present the distributions of each question in the questionnaire. The cut-offs for the three dimensions of the KAP questionnaire were based on similar studies [16].

## Results

### Population

Finally, 18 of the 90 CHS centers with a Family Medicine Residency program were randomly chosen in a 1:1:1 ratio. A total of 467 questionnaires were distributed and a total of 462 questionnaires were completed, giving a response rate of 98.93%. Participants’ ages were mainly from 40 to 49 years. Most participants had high education backgrounds. A total of 83.8% of participants had a bachelor’s degree. Most PCPs had worked over 10 years (343; 74.3%; Table 1**)**.

**Table 1:** Demographic Characteristic of PCPs

Variable	No(%)
Sex	
Male	142 (30.7)
Female	320 (69.3)
Age (years)	
aged < 30	49 (10.6)
aged 30–39	180 (39.0)
aged 40–49	192 (41.6)
aged 50–59	35 (7.6)
aged ≥60	6 (1.3)
Maximum Educational Level	
Technical School Graduate	3 (0.6)
College Degree	45 (9.7)
bachelor Degree	387 (83.8)
Master’s Degree	27 (5.8)
Types of CHS Centers	
The Urban-Rural	148 (32.0)
The Rural	158 (34.2)
The Urban	156 (33.8)
Professional Title	
Resident	90 (19.5)
Physician	326 (70.6)
Associate Senior Physician	44 (9.5)
Chief Physician	2 (0.4)
Years of Working Experience	
< 5 years	46 (10.0)
5 ~ 9 years	73 (15.8)
10 ~ 14 years	96 (20.8)
15 ~ 19 years	100 (21.6)
20 ~ 24 years	83 (18.0)
· ≥ 25 years	64 (13.9)
Attending Training	
Yes	242 (52.4)
No	220 (47.6)

### The reality and reason of anticoagulation in AF

The majority (50.6%) of patients with NVAF were 70–79 years old. With regard to OAC therapy, we found that 41.6% of PCPs treated NVAF with aspirin in more than 70% of their patients, while only 0.4% of PCPs treated NVAF with OAC in more than 70% of their patients.

**Fig. 1:**
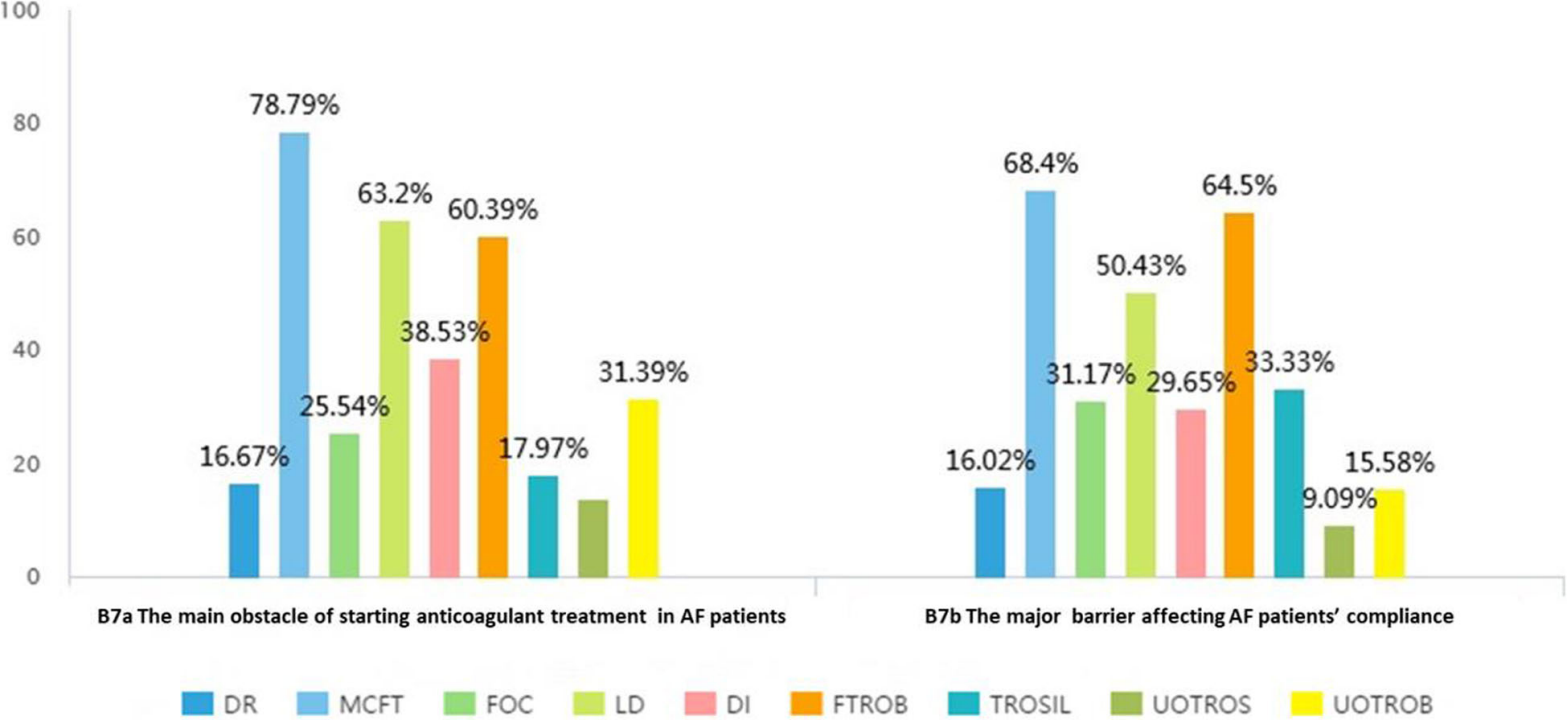
The main barriers for strating OAC therapy and affecting patients’ compliance. DR = dietary restriction. MCFT = monitoring coagulation function tests. FOC = fees of coagulation. LD = lack of drugs (medications). DI = drug-drug interactions. FTROB = fear of the risk of bleeding. TROSIL = the risk of stroke of bleeding (patients). UOTROS = unsure of the risk of stroke (doctors). UOTROS = unsure of the risk of bleeding (doctors)

Our study showed that the top three obstacles for starting OAC therapy in patients with NVAF included 1) monitoring coagulation function tests (78.79%), 2) lack of essential medicines in the community (63.20%), and 3) fear of bleeding in patients with NVAF (60.39%). The investigation also found that the first three major barriers that affected the compliance of patients with NVAF included 1) monitoring coagulation function tests (68.40%), 2) worrying about bleeding in patients with NVAF (64.50%), and 3) the lack of essential medications in CHS centers (50.43%) **(**Fig. 1**).** All the above results were based on participants’ self-assessment in the second part of the questionnaire (shown in Supplementary 1).

### Knowledge, attitudes, and practices

In this study, 4.5% of PCPs scored a “good” level of knowledge, while 75.8% had an “inadequate” level of knowledge, which meant that participants’ knowledge scores were poor or fair. The majority (89.8%) of PCPs had positive attitudes towards anticoagulation therapy in patients with NVAF; however, 19.0% had unsatisfactory practices in treating NVAF with OAC therapy **(**Table 2**)**.

**Table 2:** The scores of the KAP questionnaire (knowledge, attitude, and practice)

	Scores		
	≤39%[n(%)]	40–69%[n(%)]	≥70%[n(%)]
Knowledge	350 (75.8%)	91 (19.7%)	21 (4.5%)
Attitude	3 (0.6%)	44 (9.6%)	415 (89.8%)
Practice	88 (19.0%)	314 (68.0%)	60 (13.0%)

Table 3 shows that 62.55% (289 participants) of PCPs knew how to diagnose AF, 52.16% (241 participants) knew the frequency of the coagulation function for patients with NVAF with long-term warfarin therapy, and 38.96% (180 participants) knew the target range of INR when warfarin therapy was used in patients with NVAF who were under the age of 75 years. Nevertheless, 96.10% (444 participants) did not know which clotting indicators should apply in patients with NVAF treated with warfarin. A total of 94.8% (438 participants) of PCPs did not know the score for predicting bleeding risk in patients with NVAF, and 86.58% (400 participants) did not know of HAS-BLED, which is a bleeding risk scoring system.

**Table 3:** Community PCP knowledge of OAC therapy in NVAF patients (N = 462)

Knowledge items	yes(%)	no(%)
How to diagnose AF?	289 (62.55)	173 (37.45)
Which score tool can be used to predict stroke risk in AF patients?	112 (24.24)	350 (75.76)
Which score tool can be used to predict bleed risk in AF patients?	24 (5.19)	438 (94.81)
What risk factors does CHADS2 score include?	75 (16.23)	387 (83.77)
What risk factors does CHADS2-VASc score include?	71 (15.37)	391 (84.63)
What risk factors does HAS-BLED score include?	62 (13.42)	400 (86.58)
Which indicator should be monitored in AF patients with warfarin?	18 (3.90)	444 (96.10)
How long should be monitor coagulation function in AF patients with long-term warfarin therapy at a stable period?	241 (52.16)	221 (47.84)
What’s the target range of INR in AF patients with warfarin under 75 years old?	180 (38.96)	282 (61.04)
What’s the target range of INR in AF patients with warfarin over 75 years old?	90 (19.48)	372 (80.52)
Which factor is susceptible to the anticoagulation effect of warfarin?	159 (34.42)	303 (65.58)
What’s the antagonist that antagonize warfarin’s anticoagulation?	157 (33.98)	305 (66.02)
Which of the following AF patients need to adjust warfarin dose?	136 (29.44)	326 (70.56)
Which medication are the new oral anticoagulants (NOAC)?	86 (18.61)	376 (81.39)

The highest scoring attitude item was “to tell patients with NVAF about medication and food that affect warfarin’s anticoagulant effects,” while the lowest scoring attitude item was “more concerns about the risk of bleeding in patients with AF than the risk of stroke in patients with AF.” The scores of each item are shown in **Table S****1****.**

The highest scoring practice question was “Have you ever made a differential diagnosis based on the duration of the onset of AF when treating patients with AF?”, while the lowest scoring item was “The patient with AF in item E8 had gastrointestinal bleeding three months ago, which has stopped for 1 week. Would you give this patient oral anticoagulant therapy?” The scores of each item are shown in **Table S**2**.**

### Influence factors analysis

Using the knowledge score, attitude score, and practice score as the dependent variable, other factors were set as independent variables. According to the order of independent variable effects from large to small, the time that PCPs practiced medicine, whether the practice was outpatient, and the type of CHS center were all related to the knowledge score. Knowledge score, sex, and whether PCPs were in an inpatient ward were related to the attitudes of PCPs. Knowledge score, outpatient practice and attitude score were related to the practice score.

## Discussion

The study revealed fewer patients with NVAF who go to community clinics to seek medical attention Shanghai based on PCPs’ self-report. The reason for this finding may be due to the majority of patients with NVAF going to tertiary and secondary hospitals for treatment. Our study also found that the management of patients with NVAF in CHS centers was not standardized adequately in Shanghai. The data indicated that doctors were deficient in their knowledge of OAC therapy for NVAF, PCPs recognized the importance of OAC therapy in NVAF, and most PCPs performed poorly on the behavioral component of the questionnaire.

The specific results of the survey indicated that only 0.4% of PCPs treated their NVAF patients with warfarin anticoagulant therapy. Many other studies showed that the rate of warfarin therapy for patients with NVAF in China was less than 2% [3, 6], and Huiping Chen et al. [17] also suggested a similar conclusion in their study in China. The results of this survey are similar to those of the above studies. However, Bai Y et al. [18] showed that warfarin was superior to aspirin and antithrombotic therapy was required to reduce the risk of stroke in older patients with AF. PCPs should consider prescribing anticoagulants like warfarin to patients with NVAF in the community.

This study showed that PCPs had insufficient knowledge of anticoagulation therapy for patients with NVAF. Those findings were consistent with the results of Changing Wang et al. [4] who also observed unsatisfactory knowledge among physicians. This study found that not all PCPs knew how to diagnose AF and most PCPs did not routinely apply assessment tools to evaluate the relevant risks of patients with NVAF in their clinical practice. The low awareness of tools for stroke- and bleeding-risk calculations in patients with NVAF was noted, and we concluded that the management of OAC therapy in patients with NVAF was not ideal.

A total of 65.58% of PCPs answered questions incorrectly with regard to the factors, genes, drugs, and food that were prone to interacting with warfarin; knowledge that is essential for patient education. Therefore, most PCPs did not provide adequate levels of education to NVAF patients. The latest studies have shown that among patients with NVAF, NOAC is superior to warfarin for the prevention of AF-related strokes [19]. However, we found that 81.39% of PCPs did not know of NOAC therapy because most PCPs in the community did not have a good understanding of the literature, despite having a positive attitude toward acquiring such knowledge.

The risk of stroke and bleeding among patients with NVAF must be calculated before OAC therapy. PCPs should make the most appropriate clinical decisions after weighing the risk of bleeding against the benefit of stroke prevention in patients with NVAF. CHADS2-VASc score and HAS-BLED score are two valuable tools in making such clinical decisions; however, we found that most participants did not have a good understanding of these tools. Thus, we encourage and advise up-to-date CME among PCPs who work in the community, especially in the application of easy scoring systems to assist in superior clinical decision making. The practice score of PCPs in OAC for patients with NVAF was average; however, they had a positive attitude toward OAC. Thus, we inferred that the under-treatment of OAC in patients with NVAF was due to the lack of knowledge. Based on our questionnaire, a total of 94.4% of PCPs answered that they would participate in a specific CME program (question: if training of AF disease and anticoagulation were available, would you participate?). As their attitude was high, we suggest the establishment of an effective CME program to assist PCPs provide OAC therapy to patients with NVAF.

In multiple linear regression analysis, physicians with more years of experience and solid clinical abilities may have superior performances in managing patients with NVAF. Conversely, our study found that PCPs with fewer years of practice had higher knowledge scores. PCPs who are in inpatient settings (some CHS centers have inpatient service) had higher knowledge scores than those in outpatient settings. We found that PCPs met less patients with NVAF in outpatient clinics, and therefore, have less motivation to acquire the necessary knowledge to treat such patients. The PCPs in community hospitals in the urban and rural areas had the highest knowledge score, followed by those in community hospitals in central urban areas, with those in suburban community hospitals having the lowest knowledge scores.

The medical resources in the central urban areas are relatively abundant compared to those in the urban-rural junctions and suburbs [20]. Thus, such a factor incentivizes a large number of patients with NVAF to attend the superior hospitals for treatment. Patients in the suburbs have a poor awareness of health [21] and as a result, PCPs in downtown and suburban areas have less opportunities to manage patients with NVAF. Such a factor may explain the low knowledge scores among PCPs in the central urban and suburban regions. However, no relevant studies have been performed to discuss the reasons for the above results. The sample size of our study can be expanded to further assess such factors.

Our study also revealed the influences of attitude and behavioral scores (seen in Supplementary material 2). Based on KAP theory, individuals gradually acknowledge content when relevant information is perceived or received [16]. Therefore, PCPs’ attitudes toward OAC therapy in patients with NVAF can be changed when their need for knowledge is satisfied. Thus, selective CME training on OAC therapy can improve PCPs’ awareness for treating NVAF patients with OAC. With comprehensive care provided by PCPs to improve the rate of OAC therapy among patients with NVAF in CHS centers, we expect reductions in the morbidity of AF-related stroke, as well as improvements in the quality of life and prognosis of patients with AF.

### Strengths and limitations

To our knowledge, this was the largest cross-sectional observational study of KAPs for PCPs relating to OAC therapy in patients with NVAF. This study investigated the current conditions of OAC treatment in patients with NVAF based on PCPs’ perspectives and desires to acquire additional knowledge on the topic. This study also details comprehensive intervention measures to improve the rate of anticoagulant therapy in the community.

Due to the limited time, social desirability bias, recall bias, and resources for this study, this research can be improved further. This study included only a subset of the demographic characteristics of community PCPs and may not fully reflect the reality of the management of OAC therapy in patients with NVAF. Therefore, future studies should include multiple other factors to complement and verify the conclusions of the current study. This study is a cross-sectional survey of the current situation, and thus, intervention studies should be performed in the future to explore possible confounding factors.

## Conclusions

The knowledge and behaviors of PCPs were insufficient for OAC therapy to prevent embolization in patients with NVAF. However, the study also revealed the positive attitudes of participants, and their desire to learn the latest knowledge of OAC therapy. Thus, there is good potential for educational interventions to positively impact the care of patients with NVAF.

## Supplementary information

**Additional file 1 **Table S1 Attitude item score of community PCPs on anticoagulant therapy for NVAF patients (*n* = 462).

**Additional file 2.** Table S2 Practice item score of community PCPs on anticoagulant therapy for NVAF patients (n = 462).

**Additional file 3.**

**Additional file 4.**

**Additional file 5.**

## Data Availability

The datasets used and/or analyzed during the current study are available from the corresponding author upon reasonable request.

## Abbreviations

PCPs: Primary care physicians
OAC: Oral anticoagulants
NVAF: Non-valvular atrial fibrillation
KAP: Knowledge, attitudes and practices
CHS: Community health service
AF: Atrial Fibrillation
INR: International normal ratio
POCT: Point-of-care testing
