# Supplementary material for: A study of knowledge, attitudes, and practices of primary care physicians toward anticoagulant therapy in patients with non-valvular atrial fibrillation in Shanghai, China
Source: BMC Fam Pract. 2020 Aug 15;21:165. doi: 10.1186/s12875-020-01236-4 (PMC7429456; doi:10.1186/s12875-020-01236-4)
Supplement: Supplementary file 1 — Additional file 1 Table S1 Attitude item score of community PCPs on anticoagulant therapy for NVAF patients (n = 462). [file 12875_2020_1236_MOESM1_ESM.docx]

Table S1 Attitude item score of community PCPs on anticoagulant therapy for NVAF patients (n=462)

| Attitude Items | Min | Max | Score  （M士SD） |
| --- | --- | --- | --- |
| The type of atrial fibrillation would affect doctor’s initiate anticoagulant therapy and choose oral anticoagulants. | 1 | 5 | 3.56±1.193 |
| It is necessary to use the stroke score tool to assess the risk of stroke in AF patients before anticoagulant therapy. | 1 | 5 | 4.38±1.032 |
| It is necessary to use the bleeding score tool to assess the risk of bleeding in AF patients before anticoagulant therapy. | 1 | 5 | 4.53±0.909 |
| I am more concerned about the risk of bleeding in AF patients than the risk of stroke in AF patients | 1 | 5 | 2.52±1.183 |
| I think it's important for AF patients to "understand the risk of stroke and bleeding in patients with AF." | 1 | 5 | 4.59±0.825 |
| I think it's important for AF patients to "reduce the risk of stroke and bleeding due to atrial fibrillation." | 1 | 5 | 4.59±0.835 |
| It is safe to maintain the INR between 2.0 and 3.0 for warfarin anticoagulation therapy in NVAF patients. | 1 | 5 | 3.59±1.040 |
| It is necessary to tell AF patients about medication and food that affect warfarin's anticoagulant effects. | 1 | 5 | 4.68±0.730 |
| I fully understand the views of AF patients on reducing the risk of stroke and bleeding caused by warfarin therapy. | 1 | 5 | 4.09±0.974 |
| I think the new oral anticoagulant (NOAC) has lower risk of bleeding than warfarin. | 1 | 5 | 3.86±0.949 |
| I think the new oral anticoagulant (NOAC) is easier to administer than warfarin. | 1 | 5 | 3.85±0.933 |
| I hope to have more knowledge to discuss the advantages and disadvantages of stroke, bleeding risk and anticoagulation regimen with AF patients. | 1 | 5 | 4.54±0.786 |
| I think doctors can improve the standard anticoagulant treatment rate in AF patients after training atrial fibrillation and anticoagulation knowledge. | 1 | 5 | 4.47±0.895 |
