# Supplementary material for: A study of knowledge, attitudes, and practices of primary care physicians toward anticoagulant therapy in patients with non-valvular atrial fibrillation in Shanghai, China
Source: BMC Fam Pract. 2020 Aug 15;21:165. doi: 10.1186/s12875-020-01236-4 (PMC7429456; doi:10.1186/s12875-020-01236-4)
Supplement: Supplementary file 2 — Additional file 2. Table S2 Practice item score of community PCPs on anticoagulant therapy for NVAF patients (n = 462). [file 12875_2020_1236_MOESM2_ESM.docx]

Table S2 Practice item score of community PCPs on anticoagulant therapy for NVAF patients (n=462)

| Practice Item | Min | Max | Score  （M±SD） |
| --- | --- | --- | --- |
| Have you ever made differential diagnosis according to the duration of the onset of atrial fibrillation when you deal with AF patients? | 1 | 4 | 2.62±0.863 |
| Have you ever made differential diagnosis between valvular AF and non-valvular AF in AF patients when you deal with AF patients? | 1 | 4 | 2.07±0.930 |
| Do you use stroke risk score tools to assess stroke risk in AF patients? | 1 | 4 | 1.90±1.004 |
| Do you use bleeding risk score tools to assess bleeding risk in AF patients? | 1 | 4 | 1.85±0.973 |
| For AF patients treated with warfarin, the INR is maintained at 1.1-2.0. Would you increase the warfarin dose for this patient? | 1 | 4 | 2.04±0.964 |
| For AF patients treated with warfarin, the INR is maintained at 3.5-5.5. Would you decrease the warfarin dose for this patient? | 1 | 4 | 2.45±1.116 |
| A 75-year-old male NVAF patient, with hypertension and no history of diabetes and cardiovascular disease, would you give this patient warfarin for anticoagulant treatment? | 1 | 4 | 1.85±0.885 |
| E8 A 75-year-old female NVAF patient, with history of hypertension, congestive heart failure and TIA 3 years ago. Ultrasound indicated aortic atherosclerosis and atrial enlargement. Would you give this patient oral anticoagulant therapy? | 1 | 4 | 2.32±1.032 |
| The AF patient in E8 item had gastrointestinal bleeding 3 months ago and has stopped bleeding for 1 week. Would you give this patient oral anticoagulant therapy? | 1 | 4 | 1.41±0.712 |
| The AF patient in E8 item had nosebleeds once and gum bleeds occasionally when brushing his teeth. Would you give this patient warfarin treatment? | 1 | 4 | 1.53±0.714 |
| The AF patient in E8 item has taken coronary stent implantation for 1 month, would you give the patient dual antiplatelet and warfarin therapy? | 1 | 4 | 2.11±1.008 |
| The AF patient in E8 item with ACS has taken coronary stent implantation and has been stable for 1 year. Would you give this patient mono-antiplatelet and warfarin therapy? | 1 | 4 | 2.09±0.932 |
| A 68-year-old hypertensive female patient with recurrent episodes of paroxysmal atrial fibrillation and without previous medical history. Would you give this patient oral anticoagulant therapy? | 1 | 4 | 2.04±0.894 |
| Have you often told AF patients who use warfarin therapy about the food and drugs that interacts with warfarin? | 1 | 4 | 2.50±0.994 |
| Have you ever actively communicated with AF patients with about increasing the risk of AF-related stroke and anticoagulation therapy? | 1 | 4 | 2.28±0.845 |
| Have you ever used different methods, such as pamphlets, health lectures and education, to educate AF patients about the risk of stroke and bleeding related to AF and anticoagulant treatment? | 1 | 4 | 2.02±0.866 |
| Have you ever attended relevant training or learned lectures about atrial fibrillation diseases and anticoagulation therapy? | 1 | 4 | 1.97±0.695 |
| Will you attend the training about AF disease and anticoagulation therapy? | 1 | 4 | 2.59±0.840 |
