# Supplementary material for: A study of knowledge, attitudes, and practices of primary care physicians toward anticoagulant therapy in patients with non-valvular atrial fibrillation in Shanghai, China
Source: BMC Fam Pract. 2020 Aug 15;21:165. doi: 10.1186/s12875-020-01236-4 (PMC7429456; doi:10.1186/s12875-020-01236-4)
Supplement: Supplementary file 4 — Additional file 4. [file 12875_2020_1236_MOESM4_ESM.docx]

Supplementary 2

The influencing factors of attitude score in community PCPs regarding OAC therapy include 1) knowledge score,2) gender and 3) whether they are in the inpatient setting. PCPs with higher knowledge score have higher attitude score.

The influencing factors of behavioral score include 1) knowledge score of community PCPs on OAC therapy, 2) practice in outpatient setting, and 3) attitude score. PCPs with higher knowledge and attitude score have the higher the behavioral score.
