# Supplementary material for: A study of knowledge, attitudes, and practices of primary care physicians toward anticoagulant therapy in patients with non-valvular atrial fibrillation in Shanghai, China
Source: BMC Fam Pract. 2020 Aug 15;21:165. doi: 10.1186/s12875-020-01236-4 (PMC7429456; doi:10.1186/s12875-020-01236-4)
Supplement: Supplementary file 5 — Additional file 5. [file 12875_2020_1236_MOESM5_ESM.docx]

Supplementary3:

Knowledge-Attitude-Practice (KAP) questionnaire of community primary care physicians (PCPs) in anticoagulant therapy for non-valvular atrial fibrillation (NVAF) patients

Dear doctor,

Hello! First of all, thank you for your participation and assistance in completing this survey. This questionnaire was designed to investigate the knowledge-attitude-behaviors of community PCPs on anticoagulant therapy for NVAF patients. Since everyone has different thoughts and practices, there is no right or wrong answer. Please make the most authentic answer to this questionnaire according to your actual situation. This questionnaire is used for scientific research only. We will keep all your information confidential. Please feel free to reply.
Questionnaire coding:______ Date:_________________

Signature of investigator:_____________

I General Conditions

A1 Gender: Male=① Female=②

A2 Age: ＜30 yo=① 30～39 yo=② 40～49 yo=③ 50～59 yo=④ ≥60 yo=⑤

A3 Highest Education: technical secondary school education =①

College degree =②

Bachelor degree =③

Master degree =④

Doctor degree =⑤

A4 Types of Community Health Service (CHS) centers:

The Urban-Rural =① The Rural =② The Urban =③

A5 Professional Title:

Resident =① Physician =② Associate Senior Physician =③ Chief Physician =④

A6 Working Years:

＜5 years=① 5～10 years=② 10～15 years=③

15～20 years=④ 20～25 years=⑤ ＞25 years=⑥

A7 Attending Training: Yes=① No=②

Ⅱ The Reality and Reason of Anticoagulation in AF

B1 How many AF patients do you have in the past year?

1～9=① 10～19=② 20～49=③ 50～99=④ 100～149=⑤ ≥150=⑥ Others=⑦

B2 Which age group are your AF patients in?

＜50=① 50～59=② 60～69=③ 70～79=④ 80～84=⑤ ≥85=⑥ Others=⑦

B3 How many your AF patients take aspirin?

＜5%=① 5%～9%=② 10%～19%=③ 20%～39%=④ 40～69%=⑤ ≥70%=⑥

B4 How many your AF patients take warfarin?

＜5%=① 5%～9%=② 10%～19%=③ 20%～39%=④ 40～69%=⑤ ≥70%=⑥

Note: B5 included B5a and B5b, and the two questions share the following options (①～⑨)

B5a The main obstacle of starting anticoagulant treatment in AF patients (Multiple choices):

B5b The major barrier affecting AF patients’ compliance (Multiple choices):

dietary restricts=①

monitor coagulation function tests=②

fees of coagulation=③

lack of medications=④

drug-drug interactions=⑤

fear the risk of bleeding=⑥

the risk of stroke is low (patients)=⑦

unsure of the risk of stroke (doctors)=⑧

unsure of the risk of bleeding (doctors)=⑨

III KAP questionnaire of PCPs in anticoagulant therapy for NVAF patients

C The knowledge part of the KAP questionnaire

(The following questions are mainly about your understanding of atrial fibrillation and anticoagulation therapy. Please answer according to the actual situation. The answers can be single or multiple.)

C1 How to diagnose AF?

ECG=① Holter=② Auscultation of the heart and palpation of the pulse=③

C2 Which score tool can be used to predict stroke risk in AF patients?

CHADS2 Score=① CHADS2-VASc Score=② HAS-BLED Score=③

ORBIT Score=④ Not Known=⑤

C3 Which score tool can be used to predict bleed risk in AF patients?

CHADS2 Score=① CHADS2-VASc Score=② HAS-BLED Score=③

ORBIT Score=④ Not Known=⑤

C4 What risk factors does CHADS2 score include?

Hypertension=① Diabetes=② Dyslipidemia=③ Congestive Heart Failure=④

Female=⑤ Age＞75yo=⑥ Prior Stroke/TIA=⑦ Not Known=⑧

C5 What risk factors does CHADS2-VASc score include?

Congestive Heart Failure/Left Ventricular Dysfunction=① Hypertension=② Age≥75yo=③ Diabetes=④ Prior Stroke/TIA/Thrombosis=⑤ Vascular disease=⑥ Age 65-74yo=⑦ Female=⑧ Dyslipidemia=⑨ Not Known=⑩

C6 What risk factors does HAS-BLED score include?

Female=① Hypertension=② Liver Dysfunction and Renal Dysfunction=③

Stroke=④ History of Bleeding=⑤ Unstable INR=⑥ Alcoholism=⑦

Concomitant Medications（eg. Antiplatelet Drugs, NSAIDS）=⑧

Age＞65yo=⑨ Not Known=⑩

C7 Which indicator should be monitored in AF patients with warfarin?

PT=① APTT=② INR=③

D-Dimer=④ Fibrinogen=⑤ Not Known=⑥

C8 How long should be monitor coagulation function in AF patients with long-term warfarin therapy at a stable period?

Every 2 Days=① Every 7 Days=② Every 30 Days=③

Every 3 Months=④ Not Known=⑤

C9 What’s the target range of INR in AF patients with warfarin under 75 years old?

1.5-2.4=① 2.0-3.0=② 2.0-2.5=③

1.8-2.6=④ 2.5-3.5=⑤ Not Known=⑥

C10 What’s the target range of INR in AF patients with warfarin over 75 years old?

1.5-2.4=① 2.0-3.0=② 2.0-2.5=③

1.8-2.6=④ 2.5-3.5=⑤ Not Known=⑥

C11 Which factor is susceptible to the anticoagulation effect of warfarin?

The Patient's Genes=① Food=② Drugs=③ Not Known=④

C12 What’s the antagonist that antagonize warfarin's anticoagulation?

Vitamin K=① Protamine=② Prothrombin Complex=③

Fresh Plasma=④ Not Known=⑤

C13 Which of the following AF patients need to adjust warfarin dose？

INR 2.0-3.0=① INR 1.0-1.5=② INR 3.8-4.5=③

INR 2.0-2.5 and Age≥75yo=④ Not Known=⑤

C14 Which medication are the new oral anticoagulants (NOAC)?

Dabigatran=① Rivaroxaban=② Apixaban=③

Dicoumarin=④ Edoxaban=⑤ Not Known=⑥

D The Attitude part of the KAP questionnaire

(The following questions are set to understand your own views on atrial fibrillation and anticoagulation therapy. There is no absolute right or wrong in each view. Please choose one answer which best represents your opinion according to the topic. Choose only one best answer for each question.)

D1 The type of atrial fibrillation would affect doctor’s initiate anticoagulant therapy and choose oral anticoagulants.

Strongly Disagree=① Somewhat Disagree=② Not Sure=③

Somewhat Agree=④ Strongly Agree=⑤

D2 It is necessary to use the stroke score tool to assess the risk of stroke in AF patients before anticoagulant therapy.

Strongly Disagree=① Somewhat Disagree=② Not Sure=③

Somewhat Agree=④ Strongly Agree=⑤

D3 It is necessary to use the bleeding score tool to assess the risk of bleeding in AF patients before anticoagulant therapy.

Strongly Disagree=① Somewhat Disagree=② Not Sure=③

Somewhat Agree=④ Strongly Agree=⑤

D4 I am more concerned about the risk of bleeding in AF patients than the risk of stroke in AF patients.

Strongly Disagree=① Somewhat Disagree=② Not Sure=③

Somewhat Agree=④ Strongly Agree=⑤

D5 I think it's important for AF patients to "understand the risk of stroke and bleeding in patients with AF."

Strongly Disagree=① Somewhat Disagree=② Not Sure=③

Somewhat Agree=④ Strongly Agree=⑤

D6 I think it's important for AF patients to "reduce the risk of stroke and bleeding due to atrial fibrillation."

Strongly Disagree=① Somewhat Disagree=② Not Sure=③

Somewhat Agree=④ Strongly Agree=⑤

D7 It is safe to maintain the INR between 2.0 and 3.0 for warfarin anticoagulation therapy in NVAF patients.

Strongly Disagree=① Somewhat Disagree=② Not Sure=③

Somewhat Agree=④ Strongly Agree=⑤

D8 It is necessary to tell AF patients about medication and food that affect warfarin's anticoagulant effects.

Strongly Disagree=① Somewhat Disagree=② Not Sure=③

Somewhat Agree=④ Strongly Agree=⑤

D9 I fully understand the views of AF patients on reducing the risk of stroke and bleeding caused by warfarin therapy.

Strongly Disagree=① Somewhat Disagree=② Not Sure=③

Somewhat Agree=④ Strongly Agree=⑤

D10 I think the new oral anticoagulant (NOAC) has lower risk of bleeding than warfarin.

Strongly Disagree=① Somewhat Disagree=② Not Sure=③

Somewhat Agree=④ Strongly Agree=⑤

D11 I think the new oral anticoagulant (NOAC) is easier to administer than warfarin.

Strongly Disagree=① Somewhat Disagree=② Not Sure=③

Somewhat Agree=④ Strongly Agree=⑤

D12 I hope to have more knowledge to discuss the advantages and disadvantages of stroke, bleeding risk and anticoagulation regimen with AF patients.

Strongly Disagree=① Somewhat Disagree=② Not Sure=③

Somewhat Agree=④ Strongly Agree=⑤

D13 I think doctors can improve the standard anticoagulant treatment rate in AF patients after training atrial fibrillation and anticoagulation knowledge.

Strongly Disagree=① Somewhat Disagree=② Not Sure=③

Somewhat Agree=④ Strongly Agree=⑤

E The Practice part of the KAP questionnaire

(The following questions are designed to understand your management of anticoagulant therapy in patients with atrial fibrillation. Please choose the one answer that best fits your situation according to your normal behavior. Never=Never done this. Always=Always done this.)

E1 Have you ever made differential diagnosis according to the duration of the onset of atrial fibrillation when you deal with AF patients?

Never=① Sometimes=② Often=③ Always=④

E2 Have you ever made differential diagnosis between valvular AF and non-valvular AF in AF patients when you deal with AF patients?

Never=① Sometimes=② Often=③ Always=④

E3 Do you use stroke risk score tools to assess stroke risk in AF patients?

Never=① Sometimes=② Often=③ Always=④

E4 Do you use bleeding risk score tools to assess bleeding risk in AF patients?

Never=① Sometimes=② Often=③ Always=④

E5 For AF patients treated with warfarin, the INR is maintained at 1.1-2.0. Would you increase the warfarin dose for this patient?

Never=① Sometimes=② Often=③ Always=④

E6 For AF patients treated with warfarin, the INR is maintained at 3.5-5.5. Would you decrease the warfarin dose for this patient?

Never=① Sometimes=② Often=③ Always=④

E7 A 75-year-old male NVAF patient, with hypertension and no history of diabetes and cardiovascular disease, would you give this patient warfarin for anticoagulant treatment?

Never=① Sometimes=② Often=③ Always=④

E8 A 75-year-old female NVAF patient, with history of hypertension, congestive heart failure and TIA 3 years ago. Ultrasound indicated aortic atherosclerosis and atrial enlargement. Would you give this patient oral anticoagulant therapy?

Never=① Sometimes=② Often=③ Always=④

E9 The AF patient in E8 item had gastrointestinal bleeding 3 months ago and has stopped bleeding for 1 week. Would you give this patient oral anticoagulant therapy?

Never=① Sometimes=② Often=③ Always=④

E10 The AF patient in E8 item had nosebleeds once and gum bleeds occasionally when brushing his teeth. Would you give this patient warfarin treatment?

Never=① Sometimes=② Often=③ Always=④

E11 The AF patient in E8 item has taken coronary stent implantation for 1 month, would you give the patient dual antiplatelet and warfarin therapy?

Never=① Sometimes=② Often=③ Always=④

E12 The AF patient in E8 item with ACS has taken coronary stent implantation and has been stable for 1 year. Would you give this patient mono-antiplatelet and warfarin therapy?

Never=① Sometimes=② Often=③ Always=④

E13 A 68-year-old hypertensive female patient with recurrent episodes of paroxysmal atrial fibrillation and without previous medical history. Would you give this patient oral anticoagulant therapy?

Never=① Sometimes=② Often=③ Always=④

E14 Have you often told AF patients who use warfarin therapy about the food and drugs that interacts with warfarin?

Never=① Sometimes=② Often=③ Always=④

E15 Have you ever actively communicated with AF patients with about increasing the risk of AF-related stroke and anticoagulation therapy?

Never=① Sometimes=② Often=③ Always=④

E16 Have you ever used different methods, such as pamphlets, health lectures and education, to educate AF patients about the risk of stroke and bleeding related to AF and anticoagulant treatment?

Never=① Sometimes=② Often=③ Always=④

E17 Have you ever attended relevant training or learned lectures about atrial fibrillation diseases and anticoagulation therapy?

Never=① Sometimes=② Often=③ Always=④

E18 Will you attend the training about AF disease and anticoagulation therapy?

Never=① Sometimes=② Often=③ Always=④
